# Endothelial-Protective Actions of Diethylether Extract from *Gentiana kochiana* and Xanthone Gentiacaulein Against Oxidized LDL-Induced Injury—In Vitro Evaluation

**DOI:** 10.3390/ijms26031351

**Published:** 2025-02-05

**Authors:** Gordana Tovilović-Kovačević, Nevena Zogović, Đurđica Ignjatović, Mirko Tomić, Jelena Penjišević, Jelena Kukić-Marković, Dijana Krstić-Milošević

**Affiliations:** 1 Department of Biochemistry, Institute for Biological Research “Siniša Stanković”–National Institute of the Republic of Serbia, University of Belgrade, Bulevar Despota Stefana 142, 11108 Belgrade, Serbia; djurdjica@ibiss.bg.ac.rs (Đ.I.); mitomic@ibiss.bg.ac.rs (M.T.); 2 Department of Neurophysiology, Institute for Biological Research “Siniša Stanković”–National Institute of the Republic of Serbia, University of Belgrade, Bulevar Despota Stefana 142, 11108 Belgrade, Serbia; nevenar@ibiss.bg.ac.rs; 3 Institute of Chemistry, Metallurgy and Technology–National Institute of the Republic of Serbia, University of Belgrade, Njegoševa 12, 11000 Belgrade, Serbia; jelena.penjisevic@ihtm.bg.ac.rs; 4 Department of Pharmacognosy, Faculty of Pharmacy–University of Belgrade, Vojvode Stepe 450, 11221 Belgrade, Serbia; jelena.kukic@pharmacy.bg.ac.rs; 5 Department of Plant Physiology, Institute for Biological Research “Siniša Stanković”–National Institute of the Republic of Serbia, University of Belgrade, Bulevar despota Stefana 142, 11108 Belgrade, Serbia

**Keywords:** plant extract, xanthone aglycones, gentiacaulein, gentiakochianin, endothelial dysfunction, oxidized LDL, human EA.hy926 endothelial cells, antioxidant, anti-apoptotic, Akt/CREB/eNOS

## Abstract

Endothelial dysfunction is an early pathophysiological event in atherosclerosis. The endothelial-protective abilities of diethylether extract (EE) from the *Gentiana kochiana* (Gentianaceae) herb and its main component, xanthone aglycone gentiacaulein (GC), were evaluated in an oxidized low-density lipoprotein (oxLDL)-treated EA.hy926 endothelial cell line. The EE and GC actions were assessed using cell viability assays, flow cytometry, immunoblot, DPPH, NBT, TBARS, conjugated diene formation, and Griess tests. Both EE and GC prevented oxLDL-induced apoptosis by reducing intracellular reactive oxygen species levels, mitochondrial depolarization, and caspase activation in EA.hy926 cells. EE and GC dose-dependently diminished oxLDL-induced cellular lipid peroxidation. In cell-free conditions, EE moderately scavenged superoxide anions and had no affinity toward DPPH radicals, GC did not interact with either of investigated free radicals, while both EE and GC effectively delayed Cu²⁺-induced LDL oxidation. EE and GC upregulated oxLDL-suppressed protective Akt/CREB/eNOS and ERK signals and restored oxLDL-reduced nitric oxide levels. Therefore, EE and GC effectively counteract oxLDL-induced endothelial apoptosis by reducing oxidative stress, promoting mitochondrial recovery, and enhancing the prosurvival Akt/CREB/eNOS axis and ERK activity. Our study is the first to demonstrate that xanthone-rich EE from aerial parts of *G. kochiana* and xanthone GC alleviate oxLDL-induced endothelial cell injury, underscoring their potential for cardiovascular protection.

## 1. Introduction

Cardiovascular diseases (CVD) are a major cause of mortality worldwide, accounting for nearly a third of all deaths in 2019 according to the WHO [1]. An early pathophysiological event leading to the development of atherosclerosis, the most common CVD in developed countries, is endothelial dysfunction. This condition is characterized by an imbalance between endothelium-derived vasodilators, particularly nitric oxide (NO), and endothelium-derived vasoconstrictor factors/peptides and inflammatory cytokines, causing a shift in healthy endothelium into the procoagulant, inflammatory, and vasoconstrictor phenotype [2]. Endothelial dysfunction develops gradually as a consequence of arterial wall micro-damage caused by different genetic, environmental, and lifestyle factors, including hyperglycemia, high circulating lipid levels, insulin resistance, uremia, aging, smoking, and poor eating habits [2]. The role of high levels of low-density lipoproteins (LDL) in the onset and progression of atherosclerosis is well known, with its oxidatively modified form, oxidized LDL (oxLDL), acting as a key contributor to this process [3,4,5]. Oxidized LDL is produced from LDL in the highly oxidative environment of damaged vascular walls. It exacerbates wall damage by further reducing endothelial NO bioavailability, increasing reactive oxygen species (ROS) production, promoting inflammation and leukocyte adhesion to the endothelial layer. The following smooth muscle cell proliferation and foam cell accumulation ultimately lead to the formation of atherosclerotic plaque [5]. The final stage of this persistently disturbed state of homeostasis is plaque rupture, tissue destruction, and acute cardiovascular events (myocardial infarction, stroke). Moreover, several studies suggest that circulating levels of oxLDL can serve as a biomarker for endothelial dysfunction and the assessment of atherosclerosis severity [3,6].

Current knowledge indicates that phytochemicals, especially polyphenols found in medicinal plants and plant foods, can alleviate initial disturbance in endothelial function and modulate pathophysiological events involved in atherosclerosis progression [7,8,9]. Furthermore, clinical trials suggest an inverse relationship between dietary polyphenol intake and cardiovascular risk factors, particularly among individuals at higher risk, such as those with type 2 diabetes and hypertension [10,11]. Xanthones, yellow pigments present mainly in Gentianaceae and Guttiferae families, are a group of oxygenated heterocyclic polyphenols that exert cardiovascular-protective properties via antioxidant, anti-inflammatory, antithrombotic, and vasodilator activities [12]. Various xanthones prevent endothelial dysfunction in vitro through the activation of antioxidant Akt/Nfr2/HO-1 and the inhibition of inflammatory NFkB pathways, by the suppression of monocytes adhesion to endothelial cells, and through a decrease in proinflammatory cytokines production by endothelial cells [13,14,15]. In addition, in vivo studies show xanthones’ prominent ability to improve cardiac endothelial NO synthase (eNOS) expression and NO concentration during myocardial necrosis, decrease atherosclerotic plaque size and improve serum HDL/LDL ratio in apoE knockout mice, as well as suppress ischemic-reperfusion apoptotic/necrotic damage of cardiomyocytes in rats [16,17,18]. Ethnopharmacological studies from Italy and China document the traditional use of xanthone-containing plants for treating cardiovascular diseases, like hypertension, arrhythmias, and coronary heart disease [16,19,20].

Stemless gentian (*Gentiana kochiana* Perr. et Song., syn. *Gentiana acaulis* L.; Gentianaceae) is a species endemic to mountain areas of central and southern Europe. Root extracts of this plant are used in traditional Italian medicine as antihypertensive, antipyretic, and spasmolytic agents [19]. The most important secondary metabolites of this plant are the simple tetraoxygenated xanthones gentiacaulein (1,7-dihidroxy-3,8-dimethoxyxanthone; GC), gentiakochianin (1,7,8-trihidroxy-3-methoxyxanthone) and decussatin (1-hydroxy-3,7,8-trimethoxyxanthone), and their glycosides gentiacaulein-1-*O*-glucoside and gentiacaulein-1-*O*-primeveroside. In addition, *G. kochiana* contains secoiridoids (swertiamarin, gentiopicrin, and amarogentin), another class of secondary metabolites typical for the genus *Gentiana* [21]. Research on the antihypertensive action of the crude methanol root extract of this plant has identified xanthones gentiacaulein and gentiakochianin as accountable for plant vasorelaxant activity. Specifically, these xanthones exert an endothelium-independent ability to block calcium release from intracellular stores in vascular smooth muscle cells [22,23]. Previous studies of *G. kochiana* also revealed its significant antiglioma action in vitro and antidepressant potential in rats [24,25]. However, despite reports on the ability of xanthones to prevent endothelial dysfunction, the potential endothelial-protective effects of *G. kochiana* and its xanthones have not yet been investigated. This research aims to bridge the existing gap in studies by exploring the endothelial-protective actions of diethylether extract (EE), obtained from the aerial parts of stemless gentian, and its main component, the xanthone aglycone GC, against oxLDL-induced injury in vitro. The diethylether extract was chosen based on the high content of xanthones, consisting primarily of simple 1,3,7,8-tetraoxygenated derivatives. The protective effect of the EE and GC was evaluated using EA.hy926 endothelial cell line exposed to the high concentration of oxLDL. The influence on cell viability, mitochondrial depolarization, caspase activation, and activity of Akt and ERK protein kinases responsible for survival and endothelial homeostasis maintenance was assessed. In addition, intracellular ROS levels, ROS scavenging activity of EE and GC in cell-free conditions, as well as the impact of EE and GC on Cu^2+^-induced LDL oxidation process and oxLDL-triggered cellular lipid peroxidation were analyzed. Our results show notable endothelial-protective effects of EE and GC and provide a valuable insight into potential therapeutic strategies to prevent endothelial dysfunction.

## 2. Results

### 2.1. Identification of the Main Constituents from EE of G. kochiana

**Figure 1:**
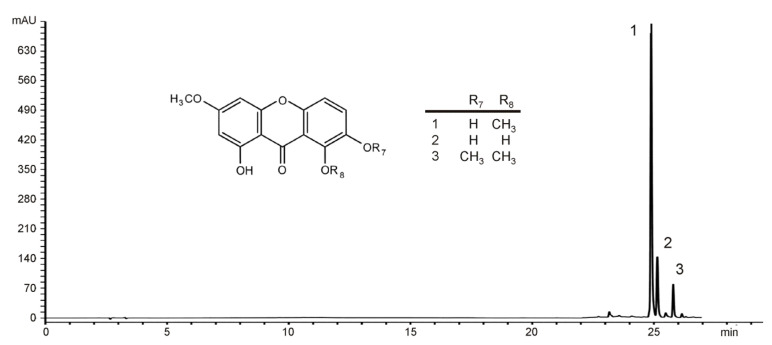
The HPLC profile of diethylether extract of *Gentiana kochiana* recorded at 260 nm and the chemical structures of the identified xanthones. Peaks: 1—gentiacaulein; 2—gentiakochianin: 3—decussatin.

HPLC analysis of the diethylether extract revealed the presence of a large amount of xanthone aglycones gentiacaulein (peak 1) and gentiakochianin (peak 2) (Figure 1). In addition, the third peak detected in the chromatogram was identified as decussatin (1-hydroxy-3,7,8-trimethoxyxanthone). Analysis of the peak areas showed that the concentrations of GC and gentiakochianin in EE were 125.74 mg/g and 17.32 mg/g, respectively. The content of decussatin was 12.55 mg/g expressed as GC equivalents/g.

### 2.2. The EE of G. kochiana and GC Protect Endothelial EA.hy926 Cells from oxLDL-Induced Death

**Figure 2:**
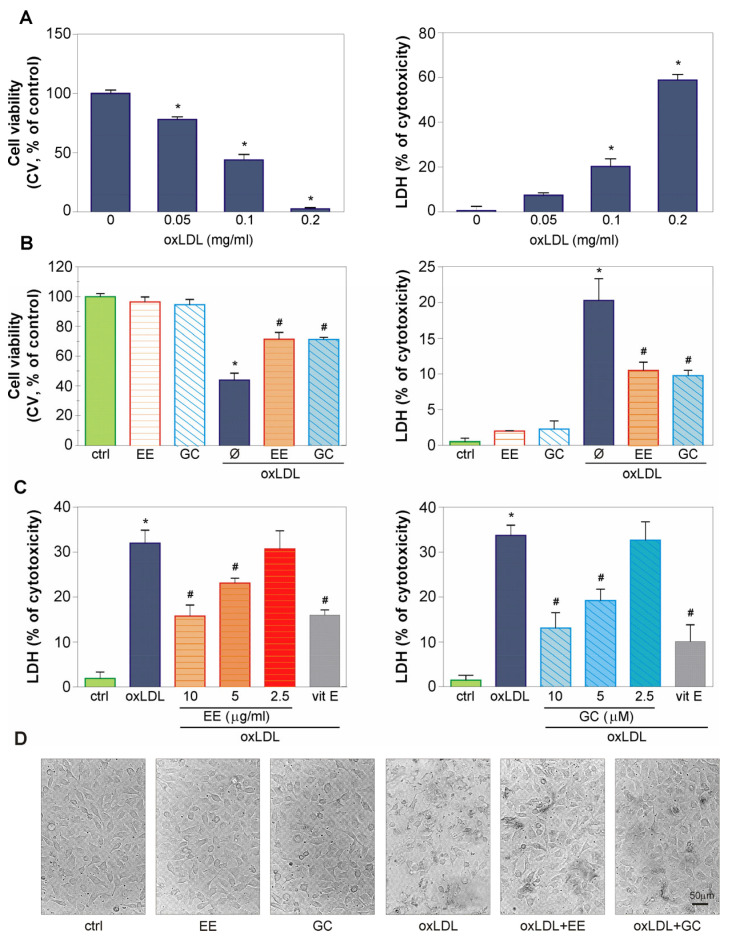
The effect of EE and GC on viability of oxLDL-treated EA.hy926 cells. Endothelial cells were incubated with different concentrations of oxLDL (0.05–0.2 mg/mL) for 48 h (**A**) or cells were pre-treated with EE (10 µg/mL) and GC (10 µM) for 30 min and exposed to oxLDL (0.1 mg/mL) for additional 48 h (**B**,**D**). Alternatively, a prior addition of oxLDL (0.1 mg/mL) cells were pre-incubated with a range of concentrations of EE and GC (as indicated in the figure) or vitamin E (50 µM) for 30 min (**C**). Cell viability was assessed using crystal violet (CV) (**A**,**B**) and lactate dehydrogenase (LDH) assays (**A**–**C**). The cell morphology was evaluated using light microscopy (**D**). The results are presented as mean ± SD values of triplicates from one representative out of three independent experiments (**A**–**C**). * *p* < 0.05 compared to control, untreated cells, # *p* < 0.05 compared to ox-LDL-treated cells.

The endothelial-protective capacity of EE and its main constituent GC was evaluated after exposure of EA.hy926 endothelial cells to the well-known endothelium damaging agent oxLDL. Crystal violet and LDH assays have shown that oxLDL dose-dependently decreased the number of adherent, viable EA.hy926 cells and increased cell membrane damage after 48 h of treatment (Figure 2A) (IC50 = 0.09 mg/mL; CV). Therefore, a concentration of 0.1 mg/mL oxLDL was chosen for further evaluation of EE and GC protective capacity. The doses of EE and GC were selected according to the range of physiologically achievable concentrations [26]. Both EE and GC significantly diminished oxLDL-induced EA.hy926 cell damage, as shown by CV and LDH assays (Figure 2B). The LDH assay demonstrated that the protective action of both EE and GC was dose-dependent (Figure 2C). The highest applied dose of EE and GC protected EA.hy926 cells as effectively as the well-known antioxidant vitamin E (Figure 2C). Additionally, light microscopy showed that EE and GC significantly prevented the oxLDL-triggered changes in cell morphology, i.e., rounding, volume reduction, detachment from the well, and subsequent reduction in the number of live, adherent EA.hy926 cells (Figure 2D).

### 2.3. The Antiapoptotic Action of EE from G. kochiana and GC Is Associated with Mitochondrial Stabilization and Caspase Inhibition

**Figure 3:**
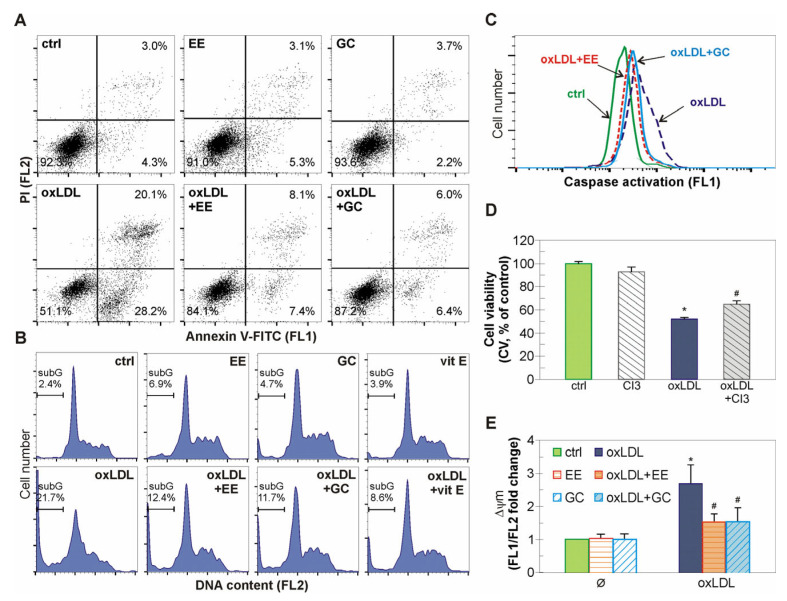
Antiapoptotic action of EE and GC in oxLDL-treated EA.hy926 cells. Antiapoptotic action of EE and GC in oxLDL-treated EA.hy926 cells. Endothelial cells were pre-incubated with EE (10 µg/mL), GC (10 µM) (**A**–**C**,**E**), and vitamin E (50 µM) (B) or caspase 3 inhibitor Z-DEVD-FMK (20 µM) (**D**) for 30 min and then treated with oxLDL (0.1 mg/mL) for 48 h (**A**,**B**,**D**) or 24 h (**C**,**E**). Flow cytometry was used to analyze the number of early and late apoptotic cells (**A**), the number of cells with hypodiploid DNA (**B**), caspase activity (**C**), and mitochondrial membrane potential (**E**) after staining cells with Annexin-V-FITC/PI (**A**), PI (**B**), ApoStat (**C**), and JC-1 (**E**). The cell viability was assessed using CV assay (**D**). The results are presented as representative dot plots (**A**), histograms (**B**,**C**), mean ± SD values of triplicates (**D**) from one representative out of three independent experiments, or as mean ± SD values of fold increase in JC-1 fluorescence intensity (**E**) from two independent experiments. * *p* < 0.05 compared to control, untreated cells, # *p* < 0.05 compared to ox-LDL-treated cells.

We further evaluated the type of oxLDL-induced cell death in EA.hy926 cells. The well-known pro-apoptotic effect of oxLDL on endothelial cells was confirmed by flow cytometric analysis of cells double stained with the fluorescent dyes Annexin V-FITC and PI [27,28]. Treatment with oxLDL increased the number of early and late apoptotic events, as judged by the elevation in the number of phosphatidylserine-exposed Ann^+^PI^−^ cells and Ann^+^PI^+^ cells with ruptured membranes, respectively (Figure 3A). Both EE and GC effectively restored the number of live, undamaged (Ann^−^PI^−^) endothelial cells to near control levels (Figure 3A). Accordingly, the flow cytometry of PI-labeled cells revealed that oxLDL caused an accumulation of hypodiploid cells with fragmented DNA in the subG region of the cell cycle. The increase in the level of these likely apoptotic cells was significantly diminished by the pre-treatment with EE and GC (Figure 3B). A similar effect was detected in the presence of the well-known antioxidant vitamin E (Figure 3B). Since caspases can mediate apoptotic cell death, we next investigated the activity of these cellular proteases using fluorescent probe ApoStat. The flow cytometry of ApoStat-labeled cells showed that EE and GC partially abrogated the oxLDL-triggered activation of caspases (Figure 3C). The caspase-dependent nature of the pro-apoptotic oxLDL stimulus was further confirmed by the partial restoration of EA.hy926 cells viability in the presence of a caspase-3 inhibitor (Figure 3D). Since caspase activation is usually a consequence of mitochondrial damage, we next evaluated the mitochondrial membrane potential in the cells stained with mitochondrial dye JC1. The flow cytometry results confirmed a marked decrease in mitochondrial membrane potential induced by oxLDL, which was partially restored by pre-treatment with EE and GC (Figure 3E). Overall, these results suggest that EE and GC counteract oxLDL-induced caspase-dependent apoptosis in endothelial cells probably by maintaining mitochondrial membrane stability.

### 2.4. The EE of G. kochiana and GC Decrease Intracellular ROS Accumulation and Inhibit Lipid Peroxidation in oxLDL-Treated Endothelial Cells

**Figure 4:**
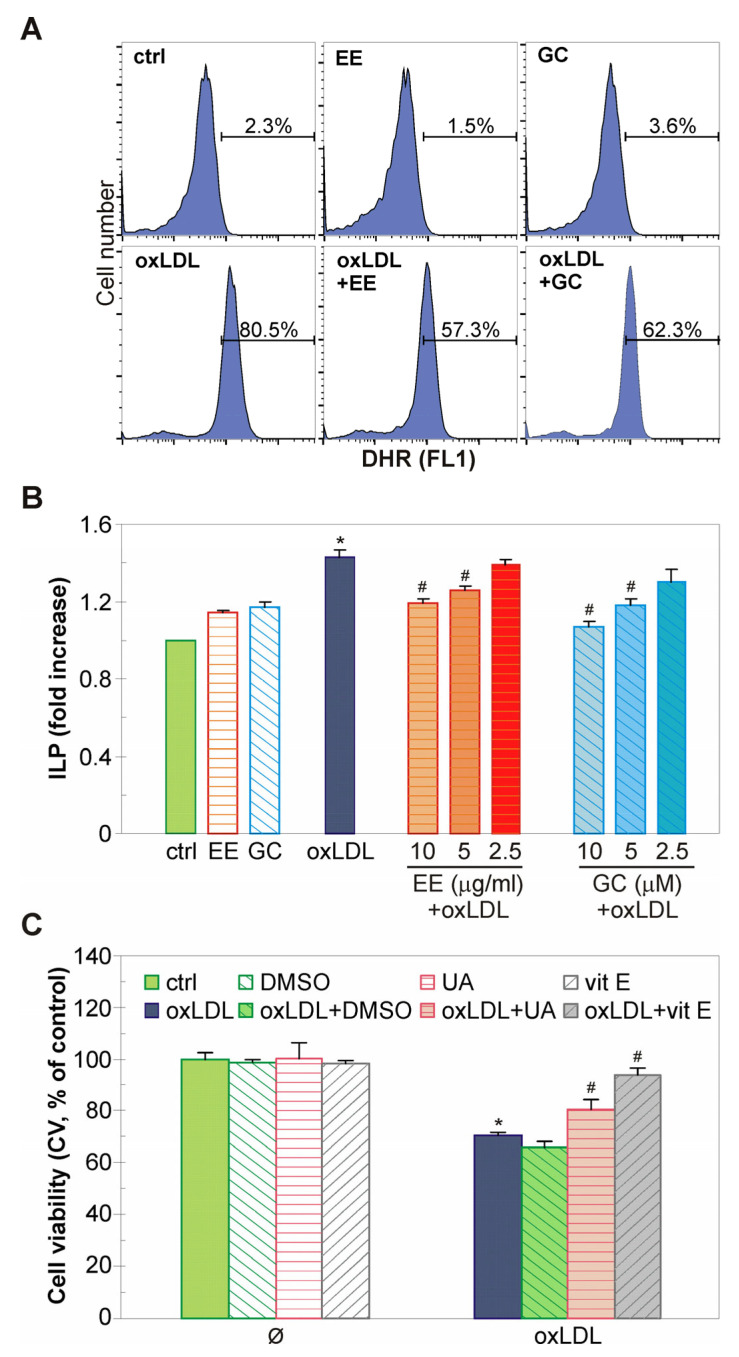
Antioxidant activity of EE and GC in EA.hy926 cells. The EA.hy926 cells were pre-treated with EE (10 µg/mL) and GC (10 µM) (**A**,**B**), DMSO (0.5%), uric acid (100 µM), vitamin E (50 µM), or with different concentrations of EE and GC (as indicated in the Figure) (**B**) for 30 min and incubated with oxLDL (0.1 mg/mL) for an additional 24 h (**A**) or 48 h (**B**,**C**). Green fluorescence of DHR-stained cells indicating intracellular levels of total ROS were determined by flow cytometry (**A**). The levels of MDA, reflecting index of lipid peroxidation in EA.hy926 cells, were measured using TBARS assay (**B**), while cell viability was assessed using CV assay (**C**). The results are presented as representative histograms (**A**) or mean ± SD values of triplicates (**B**,**C**) from one representative out of two independent experiments. * *p* < 0.05 compared to control, untreated cells, # *p* < 0.05 compared to ox-LDL-treated cells.

The damaging effect of oxLDL is based on its ability to increase the concentration of harmful ROS in endothelial cells [27,28]. Therefore, flow cytometry of DHR-loaded cells was employed to assess the influence of EE and GC on intracellular ROS concentration in oxLDL-treated cells. The results have shown a partial suppression of an oxLDL-triggered increase in ROS levels in the presence of EE and GC (Figure 4A). Next, the capacity of EE and GC to suppress oxLDL-induced oxidative damage in endothelial cells was examined by measuring the lipid peroxidation index (ILP). The TBARS assay has revealed that EE and GC dose-dependently reduced the oxLDL-induced formation of malondialdehyde, a fatty acid peroxidation product, in EA.hy926 cells (Figure 4B). To discern a possible mechanism of oxLDL-mediated oxidative damage, EA.hy926 cells were pretreated with several well-known antioxidants prior to oxLDL exposure. As crystal violet test has shown, the hydroxide radical scavenger DMSO did not protect endothelial cells; the singlet oxygen, peroxyl radical, hydroxyl radical, and peroxynitrite scavenger uric acid partially protected, while the lipid peroxidation inhibitor vitamin E restored EA.hy926 cells viability to near control levels (Figure 4C). These results imply that EE and GC are able to reduce intracellular ROS levels and suppress lipid peroxidation in oxLDL-loaded endothelial cells.

### 2.5. The EE of G. kochiana and GC Do Not Exert Antiradical Activity, but Delay Cu^2+^-Induced Oxidation of LDL Particles

**Figure 5:**
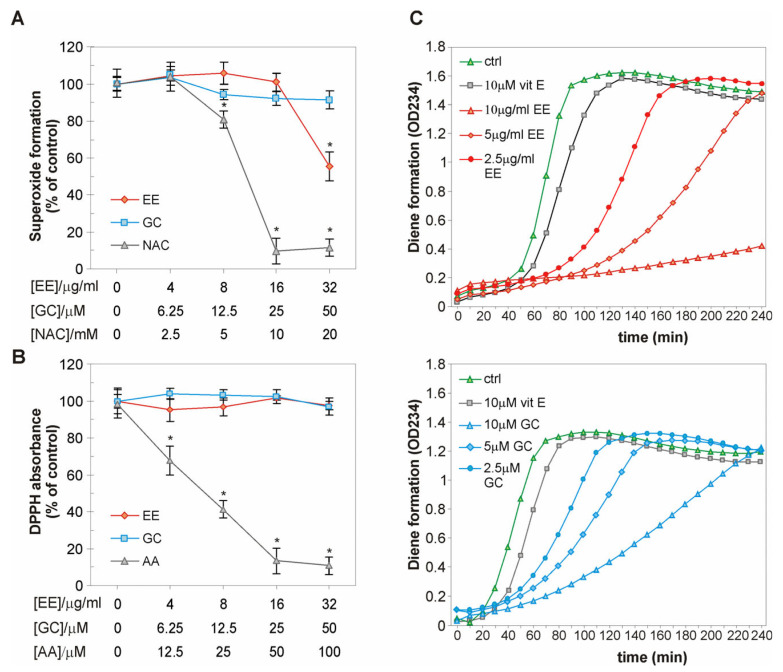
Antiradical activity of EE and GC in cell-free conditions. Different concentrations of EE and GC (**A**,**B**) or standard antioxidants NAC (**A**) and AA (**B**) were incubated with alkaline DMSO (**A**) or free DPPH radical (50 µM) (**B**). The absorbance was detected by spectrophotometry at 560 nm (**A**) or 517 nm (**B**). Alternatively, EE, GC, and vitamin E (**C**) were mixed with LDL (0.1 mg/mL) and CuSO_4_ (10 µM) and incubated for 4 h. Formation of conjugated dienes was monitored every 10 min spectrophotometrically at 234 nm (**C**). The data are presented as mean ± SD values of triplicates from one representative out of three independent experiments (**A**,**B**) or as representative curves from one out of two independent experiments (**C**). * *p* < 0.05 compared to control.

To test whether the reduction in ILP and oxidative stress is a consequence of the ROS-scavenging properties of EE and GC, we evaluated their ability to reduce the concentration of two well-known free radicals, superoxide anion (O_2_^•−^) and DPPH, in cell-free assays. The NBT test has shown that GC did not scavenge O_2_^•−^ generated by alkaline DMSO, while EE exhibited moderate potential to eliminate O_2_^•−^ (IC50(EE) = 66.1 µg/mL; IC50(GC) = n.d.; IC50(NAC) = 6.4 mM) (Figure 5A). The DPPH assay showed that EE and GC were unable to eliminate the stable DPPH free radical (IC50(EE, GC) = n.d.; IC50(AA) = 19.6 µM) (Figure 5B). Considering the well-known ability of xanthones to interfere with the LDL oxidation process, we next evaluated the influence of EE and GC on Cu^2+^-induced LDL oxidation [29,30]. EE and GC delayed the formation of conjugated dienes even more effectively than the standard antioxidant vitamin E (Figure 5C, Table 1). Collectively, these results indicate that the antioxidant effect of EE and GC is probably not due to a direct scavenging capacity but may be related to their ability to interfere with the LDL oxidation process.

### 2.6. The Endothelial-Protective Action of EE and GC Is Associated with Reactivation of the Akt/CREB/eNOS Axis, ERK Kinase, and Restoration of NO Levels in oxLDL-Treated Endothelial Cells

**Figure 6:**
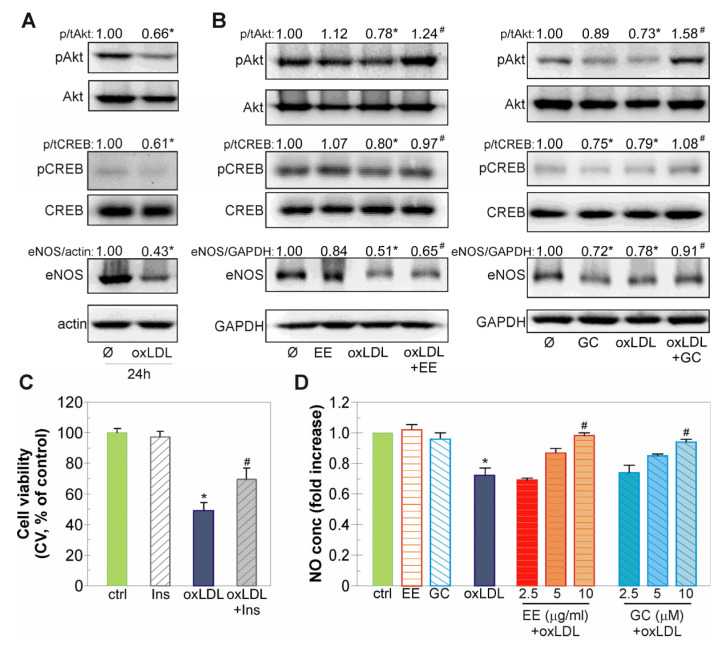
EE and GC restore Akt/CREB/eNOS axis activity and NO levels in oxLDL-treated EA.hy926 cells. The endothelial cells were exposed to oxLDL (0.1 mg/mL) for 24 h (**A**). Alternatively, the cells were pre-treated with EE (10 µg/mL), GC (10 µM) (**B**), insulin (120 nM) (**C**), or different concentrations of EE and GC (as indicated in the figure) (**D**) for 30 min and oxLDL (0.1 mg/mL) was added for additional 24 h (**B**) or 48 h (**C**,**D**). The activity of Akt and CREB and the levels of eNOS protein were analyzed by immunoblot (**A**,**B**). Actin and GAPDH were used as loading controls. Cell viability was assessed by CV test (**C**), while nitrite concentration, reflecting the amount of NO, was determined using Griess assay (**D**). The results are presented as representative blots from one out of three independent experiments (**A**,**B**) or as mean ± SD values of triplicates (**C**,**D**) from one representative out of two independent experiments. * *p* < 0.05 compared to control, untreated cells, # *p* < 0.05 compared to ox-LDL-treated cells.

Oxidized LDL modulates the activity of various intracellular kinases involved in endothelial cell death, such as Akt, ERK, and AMPK [31,32,33]. Immunoblot analysis showed that oxLDL significantly decreased the phosphorylation of the protective kinase Akt and its downstream targets CREB and eNOS (Figure 6A). EE and GC restored the oxLDL-triggered downregulation of Akt/CREB/eNOS axis activity (Figure 6B). Interestingly, EE alone had no effect on the phosphorylation of CREB and eNOS levels, whereas treatment with GC alone slightly but significantly decreased the ratio of p/tCREB and eNOS/GAPDH (Figure 6B). The detected Akt inhibition is at least partly responsible for the deleterious effects of oxLDL, as the pharmacological Akt activator insulin partially recovered the viability of oxLDL-treated EA.hy926 cells (Figure 6C). Since xanthones are able to restore the content of important vasodilator molecule NO in endothelial cells, we next investigated the effect of EE and GC on the production of NO in the conditioned medium of EA.hy926 cells treated with oxLDL [14,32]. The investigated extract and compound restored the oxLDL-induced reduction in NO levels in a dose-dependent manner (Figure 6D).

**Figure 7:**
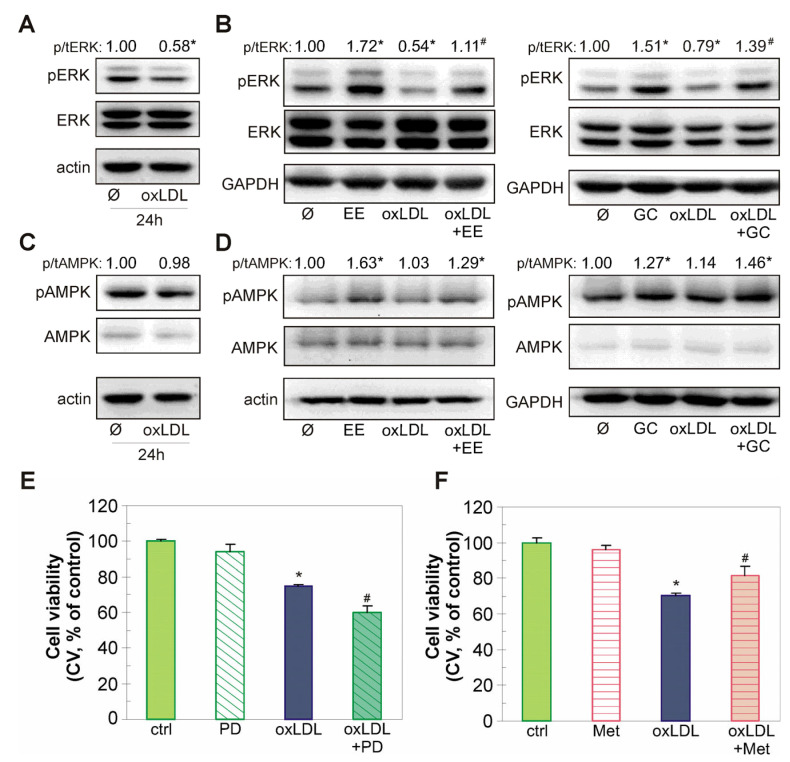
The effect of EE and GC on the activity of intracellular ERK and AMPK kinases. The EA.hy926 cells were exposed to EE (10 µg/mL), GC (10 µM) (**B**,**D**), ERK inhibitor PD98059 (10 µM) (**E**), or AMPK activator metformin (4 mM) (**F**) for 30 min and then treated with oxLDL (0.1 mg/mL) for 24 h (**B**,**D**) or 48 h (**E**,**F**). Alternatively, cells were exposed to oxLDL (0.1 mg/mL) for 24 h (**A**,**C**). The activation of intracellular kinases ERK and AMPK was determined by immunoblot (**A**–**D**). Equal protein loading was monitored using actin or GAPDH antibodies. Cell viability was determined using CV test (**E**,**F**). The data are presented as representative blots from one out of three independent experiments (**A**–**D**) or as mean ± SD values of triplicates (**E**,**F**) from one representative out of three independent experiments. * *p* < 0.05 compared to control, untreated cells, # *p* < 0.05 compared to ox-LDL-treated cells.

In addition, oxLDL markedly diminished activity of the endothelial cell homeostasis mediator ERK (Figure 7A). Both EE and GC effectively prevented this oxLDL-induced downregulation of ERK, while simultaneously increasing ERK kinase activity in EA.hy926 cells not exposed to oxLDL (Figure 7B). The activity of the energetic sensor AMPK was not affected by oxLDL (Figure 7C), but EE and GC increased the phosphorylation of this kinase both alone and in combination with oxLDL (Figure 7D). Accordingly, the MEK/ERK inhibitor PD98059 exacerbated the deleterious effect of oxLDL in endothelial cells (Figure 7E), whereas the well-known AMPK activator metformin partially recovered viability of EA.hy926 cells exposed to oxLDL (Figure 7F). Taken together, these data imply that EE and GC counteract oxLDL-induced endothelial cell injury by reactivating Akt/CREB/eNOS and ERK signaling pathways and restoring NO levels.

## 3. Discussion

In this study, we show, for the first time, that xanthone-rich EE from aerial parts of stemless gentian and its main constituent xanthone GC alleviate oxLDL-induced EA.hy926 endothelial cell injury. The EE and GC suppress the proapototic action of oxLDL by preventing mitochondrial depolarization, diminishing intracellular oxidative stress, and by recovering the activity of survival and homeostasis-related intracellular Akt/CREB/eNOS signaling pathway and ERK kinase. Additionally, EE and GC show the potential to restore the oxLDL-impaired levels of endothelial vasodilator molecule NO.

The ability of polyphenols, including xanthones, to protect endothelial cells from lipid-directed cell death in vitro and in vivo is well documented [13,32,34]. They exert protective effects on lipids-challenged endothelial cells through oxidative stress alleviation via anti-inflammatory actions and/or by restoring levels of vasodilatory molecule NO. In line with numerous other studies, oxLDL, used in this study as a pro-atherogenic lipid, increased intracellular ROS levels, significantly reduced mitochondrial membrane potential, and triggered caspase-dependent apoptosis of endothelial EA.hy926 cells [27,28]. Moreover, both EE and GC exerted an endothelial-protective action by decreasing all followed apoptosis hallmarks, i.e., mitochondrial depolarization, phosphatidylserine exposure, caspase activation, and subsequent DNA fragmentation. The restoration of mitochondrial potential by EE and GC is consistent with the findings on the capacity of other xanthones, mangiferin, and alpha-mangostin to stabilize mitochondrial membrane and protect human dermal fibroblasts, aortic endothelial cells, and primary hepatocytes from an oxidative insult triggered by H_2_O_2_ and free-fatty acids overload, respectively [35,36,37]. Mitochondrial potential recovery mediated by EE and GC can further stabilize mitochondrial membrane and prevent the release of caspase-activating molecules, a notion in agreement with the study by Pardo-Andreu et al. (2008) who showed that the xanthone mangiferin reduced mitochondrial susceptibility to permeability transition in atherosclerosis-prone mice [38]. In addition to the mitochondria-stabilizing effect, a ROS level decline was detected in EE- and GC-treated cells. A vicious interplay between elevated ROS and mitochondrial dysfunction exists in numerous pathophysiological conditions including atherosclerosis, with increased intracellular ROS inducing mitochondrial depolarization and vice versa [39]. Therefore, the observed result may either reflect the ability of EE and GC to reduce the initial oxLDL-driven intracellular ROS increase, or it could stem from mitochondrial stabilization and subsequent reduction in ROS accumulation. Further research is needed to determine the exact contribution of each of these two presumable, non-restrictive mechanisms to the protective effect of EE and GC.

The impact of EE and GC on ROS levels can be connected with the well-documented antioxidant nature of polyphenol-based xanthones, so we have evaluated the scavenging activity of EE and GC in cell-free assays [40,41]. Our results indicate that EE and GC at physiological (lower than 10 µg/mL or 10 µM) or supraphysiological concentrations do not have significant capacity to scavenge DPPH radicals. This is in line with other studies on the antioxidant activity of simple oxygenated xanthones containing 1,7-dihydroxy moiety [42]. The slightly increased ability of EE to neutralize the O_2_^•−^ anion radical, compared to GC, implies the presence of additional compounds in the investigated extract that are able to interact with the O_2_^•−^ anion radical, such as the second abundant aglycone identified in EE—trihydroxylated xanthone gentiakochianin. However, since we used a lower concentration range for EE and GC in our cell-based assays, free radical quenching does not appear to be the likely mechanism underlying their protective activity. This notion is consistent with the inability of other ROS scavengers, like DMSO and uric acid, to protect EA.hy926 cells from oxLDL-induced injury (Figure 4C). In addition, oxLDL significantly increased ILP, while vitamin E, a liposoluble antioxidant that can prevent or slow lipid peroxidation, was most effective in suppressing oxLDL-induced apoptosis of EA.hy926 cells. Therefore, it could be speculated that the lipid peroxidation chain reaction is one of the important oxidation events mediating toxicity of oxLDL in our study, while the prevention of lipid peroxidation by protective agents is responsible for endothelial cell survival. Accordingly, the ability of EE and GC to reduce the levels of MDA, an end product of lipid peroxidation, in oxLDL-treated endothelial cells strongly suggests their lipid peroxidation inhibitory effect. This assumption is also supported by the marked potential of EE and GC to delay Cu^2+^-induced formation of conjugated dienes during LDL peroxidation observed here and in numerous other scientific reports, which show that simple oxygenated and prenylated xanthones strongly inhibit oxidation of cellular lipids and LDL particles [14,29,30]. Effective antioxidants prevent lipid peroxidation through direct interaction with lipid peroxyl radicals or indirectly by quenching free radicals and chelating metal ions [43]. As EE and GC strongly delayed Cu^2+^-induced LDL peroxidation, without exhibiting significant scavenging activity, one explanation could be that they interfere with the peroxidation of cellular lipids by acting as metal ion chelating agents. The presumed ability of GC to chelate metal ions without scavenging DPPH can be correlated with the type and orientation of substituents near the carbonyl group (methoxy at C8, hydroxy at C1), which would enable metal chelation, but hinder the scavenging capacity of this xanthone [44]. On the other hand, EE and GC could interact with the lipid peroxyl ROO^•^ radical and neutralize it, as suggested for other xanthone derivatives [45]. It is important to note that EE and GC did not protect endothelial cells from damage induced by other oxidants (homocysteine, menadione, hydrogen peroxide) (Figure S1), suggesting a selective action against lipid peroxidation inducers.

Restoration of the activity of two important survival intracellular kinases, Akt and ERK, is associated with the anti-apoptotic effect of EE and GC in oxLDL-treated endothelial cells. Both Akt and ERK play a significant role in maintaining endothelial homeostasis and protecting vascular wall cells from oxidative stress and apoptosis in various atherosclerosis models in vitro and in vivo [9,32,46,47,48]. The anti-atherosclerotic role of Akt is associated with the upregulation of eNOS activity and transcription factors associated with antioxidant defense (e.g., CREB), as well as with modulation of the activity of apoptosis-related molecules in endothelial cells exposed to various atherosclerotic stimuli [46,49,50]. Similarly, the MEK/ERK signaling pathway inhibits pro-apoptotic kinases in monocytes treated with 7-ketocholesterol, a major oxidation product of cholesterol in human atherosclerotic plaques, and enhances antioxidant capacity of HUVEC endothelial cells in a model of diabetic atherosclerotic pathology [51,52]. Consistent with these studies, EE and GC restored the Akt/CREB/eNOS axis activity in EA.hy926 cells treated with oxLDL and reversed attenuated NO levels nearly to those detected in untreated cells. Furthermore, EE- and GC-driven recovery of ERK function in oxLDL-exposed cells suggests that it plays a role in protection provided by extract and xanthone. Such an assumption is indirectly supported by the observation that pharmacologic ERK inhibition augments the deleterious effects of oxLDL in EA.hy926 cells. It has been reported that similar restoration of Akt and ERK activity by the polyphenol flavanonol dihydromyricetin mediates its protective effect against oxLDL damage in HUVEC cell cultures [31]. In addition, the naturally occurring xanthones mangiferin and demethylbellidifolin attenuated oxLDL-triggered injury by increasing Akt and eNOS activity in endothelial cells and restoring serum and cellular NO levels impaired by oxLDL [14,31,32]. In line with these studies, our results suggest that EE and GC may enhance NO bioavailability and thereby improve endothelial function. It should be emphasized that, despite a slight decrease in CREB phosphorylation and eNOS levels, GC effectively prevented oxLDL-driven changes in the activity of these two downstream Akt targets. This suggests that GC alone and especially as a component of EE has the ability to reverse lipid-induced damage to endothelial cells. In addition, although oxLDL did not affect the activity of AMPK, an important energy-sensing kinase, EE and GC increased its activation both alone and in oxLDL-treated cells. Several lines of evidence indicate that enhancement of AMPK activity by various phytocompounds attenuates endothelial cell dysfunction caused by oxLDL [33,49]. In view of this, and given the previously demonstrated interaction between AMPK and Akt activity and NO production in endothelial cells, the observed activation of AMPK by xanthones in this experimental setting deserves further attention [49,53].

Our results indicate that the simple tetraoxygenated xanthone GC is likely a main active component of EE. This is supported by the ability of GC to mimic the effects of EE on cell viability, apoptosis inhibition, oxidative stress reduction, and modulation of Akt/CREB/eNOS, ERK, and AMPK activity. Additionally, HPLC analysis shows that GC is the most abundant xanthone component of EE. Gentiakochianin, another xanthone aglycone identified in significant amounts in EE, was not examined in this study due to the limited amount isolated, but its structural similarity to GC suggests that it may also contribute to the protective effects of EE. Indeed, the antihypertensive properties of *G. kochiana* on aortic rings pre-contracted with norepinephrine, KCl, or caffeine have been attributed to both GC and gentiakochianin [23]. Another important consideration regarding the physiological relevance of our findings is the potential bioavailability of the extract’s components, particularly GC, after oral administration. To assess this, we analyzed the physicochemical descriptors that can predict pharmacokinetic properties using the SWISS ADME software (http://www.swissadme.ch). According to the calculations, GC adheres to all of Lipinski’s rules of five for potentially bioavailable active compounds after oral administration: a molecular weight below 500 (Mw = 288.25), less than 10 hydrogen bond acceptors (HBA = 6), less than 10 hydrogen bond donors (HBD = 2), and a calculated octanol-water partition coefficient below 5 (LogP = 2.54). Although we can assume that GC can reach physiologically relevant concentrations required to interact with its biological targets, further evaluation of its bioavailability, therapeutic effect, and safety is necessary.

## 4. Materials and Methods

### 4.1. Plant Material and HPLC Analysis

*G. kochiana* was collected in 2015 during the flowering period on Mount Komovi (2000 m). The aerial parts of the plant were air-dried and extracted with methanol for 48 h at room temperature. The methanol extract was filtered and evaporated to dryness in a vacuum rotary evaporator (Buchi R-210, Flawil, Switzerland) at 50°. The dry extract was redissolved in distilled water and separated with solvents of increasing polarity (diethyl ether, ethyl acetate, and N-butanol) through a separatory funnel. The extracts obtained were evaporated and analyzed using the HPLC method.

The isolation, identification, and characterization of xanthones gentiacaulein, gentiakochianin, and decussatin have been reported in previous studies [21,25]. Identification of GC and gentiakochianin was confirmed using the HPLC co-injection method. Xanthone decussatin was identified by comparing the UV spectral data with reference data from our previous study [23]. HPLC analysis was performed using an Agilent 1100 series instrument with diode array detector (Agilent Technologies, Waldronn, Germany). Xanthones were separated on a Zorbax SB-C18 (Agilent Technologies, Waldronn, Germany) reversed-phase analytical column (250 mm × 4.6 mm i.d., 5 μm particle size) thermostatted at 25 °C. The mobile phase, consisting of 0.1% orthofosforic acid in water (A) and acetonitrile (B) (J.T. Baker, Deventer, The Netherlands), was eluted according to a gradient elution program as follows: 90–85% A 0–3 min, 85–70% A 3–18 min, 70–0% A 18–23 min, 0% A 23–27 min.

The injection volume of the samples was 5 μL. The detection wavelengths were set to 260 and 320 nm, and the flow rate was 1 mL min^−1^. The contents of xanthones GC and gentiakochianin were determined using calibration curves in the external standard method, while the content of decussatin was quantified as equivalents of gentiacaulein. The results are expressed as mg/g of dry extract.

### 4.2. Cell Culture and Treatments

The human endothelial cells EA.hy926 were obtained from the American Type Culture Collection (ATCC, Manassas, VA, USA). The culture was maintained in Dulbecco’s Modified Eagle Media supplemented with 10% fetal bovine serum, 2 mM L-glutamine, 100 µg/mL streptomycin, 100 U/mL penicillin (all from Sigma-Aldrich, St. Louis, MO, USA), and 20% HAT media supplement (Thermo Fisher Scientific, Waltham, MA, USA) and incubated at 37 °C in a humidified atmosphere with 5% CO_2_. Cells were prepared for experiments using the conventional trypsinization procedure with trypsin/EDTA and seeded in 96-well flat-bottom plates (1 × 10^4^ cells/well) for cell viability assays, in 6-well plates (2.5 × 10^5^ cells/well) for flow cytometric analysis, or in 60 mm Petri dishes (1 × 10^6^ cells) for immunoblotting. Cells were allowed to rest in cell culture medium for 24 h and then treated with EE, GC, and oxLDL in the presence or absence of vitamin E (vit E), dimethyl sulfoxide (DMSO), uric acid (UA), ascorbic acid (AA), N-acetyl-cysteine (NAC), the caspase-3 inhibitor Z-DEVD-FMK (CI3), insulin (Ins), the MEK/ERK inhibitor PD98059, and the AMPK activator metformin (Met) as described in Results and Figure legends. All chemicals used in this study were from Sigma-Aldrich (Sigma-Aldrich, St. Louis, MO, USA) unless otherwise stated.

The EE (20 mg/mL) and GC (20 mM) were kept as stock solutions in dimethyl sulfoxide at 4 °C until use and diluted in the cell culture medium prior treatment.

### 4.3. Oxidation of Low-Density Lipoprotein

Low-density lipoprotein (purchased from Merck, Darmstadt, Germany) was exposed to CuSO_4_ (10 µM) at 37 °C overnight and oxidation was stopped with EDTA (0.3 mM). Oxidized LDL was stored at 4 °C and used within 2 weeks. The oxidation process was verified using TBARS assay.

### 4.4. Cell Viability

The number of adherent cells and the activity of the intracellular enzyme lactate dehydrogenase released in the medium, as indicators of cell viability and oxLDL cytotoxicity, respectively, were assessed by crystal violet (CV) and lactate dehydrogenase (LDH) assay. Non-adherent dead cells were removed by washing with PBS for the CV test. The adherent, viable cells were then fixed with methanol and stained with a 10% CV solution. After a 15 min incubation at room temperature, the cultures were thoroughly rinsed with water, and the CV dye bound to the adherent cells was dissolved in 33% acetic acid. The absorbance of the dissolved dye, reflecting the number of adherent (viable) cells, was measured at 570 nm using an automated microplate reader (Multiskan Spectrum, Thermo Fisher Scientific, Waltham, MA, USA). The results of the CV assays were expressed as a percentage of viability compared to untreated control cultures, which were considered 100% viable.

The release of the cytosolic enzyme LDH in cell culture medium, a marker of cell membrane damage, was determined by incubating equal volumes of medium and LDH substrate (54 mM lactic acid, 0.66 mM p-iodonitrotetrazolium violet, 0.28 mM phenazine methosulfate, and 1.3 mM β-NAD) (all from Sigma-Aldrich, St. Louis, MO, USA) for 10 min. Pyruvate-mediated conversion of 2,4-dinitrophenylhydrazine to a visible hydrazone precipitate, corresponding to the number of dead cells, was measured using an automated microplate reader at 492 nm (Multiskan Spectrum, Thermo Fisher Scientific, Waltham, MA, USA). The percent of cytotoxicity was calculated using the following formula: [(E − C)/(T − C)] × 100, where E is the absorbance of treated cells, C is the control absorbance of untreated cells, and T is the absorbance corresponding to the maximum (100%) LDH release of cells lysed with Triton X-100.

### 4.5. Light Microscopy

The brightfield images of EA.hy926 cells were acquired with a Bio-Rad ZOE cell imager (Bio-Rad Laboratories, Hercules, CA, USA).

### 4.6. Detection of Apoptosis by Annexin V-FITC/PI Double Staining

The type of cell death was determined by double staining the cells with Annexin V-FITC (BD Biosciences, Heidelberg, Germany) and propidium iodide (PI, Sigma-Aldrich, St. Louis, MO, USA), according to the manufacturer’s recommendations. Green (FL1) and red (FL2) fluorescence was measured using a BD FACSAria III flow cytometer equipped with BD FACSDiva™ software v9.0 (BD Biosciences, San Jose, CA, USA) to evaluate the number of viable (Ann^−^/PI^−^), early apoptotic (Ann^+^/PI^−^), and late apoptotic/necrotic cells (Ann^+^/PI^+^).

### 4.7. Cell Cycle Analysis

To analyze the distribution of cells across different phases of the cell cycle, as well as hypodiploid cells with fragmented DNA (sub-G_0_/G_1_), the detached cells were stained with PI and then analyzed using a BD FACSAria III flow cytometer.

### 4.8. Caspase Activation

To assess the activity of caspases, the cysteine proteases involved in apoptotic cell death, detached cells were stained with the pan-caspase inhibitor ApoStat (R&D Systems, Minneapolis, MN, USA) according to the manufacturer’s guidelines. The increase in green fluorescence (FL1), indicative of caspase activation, was measured using a BD FACSAria III flow cytometer (BD Biosciences, San Jose, CA, USA).

### 4.9. Mitochondrial Membrane Potential Assessment

Mitochondrial membrane potential was evaluated using JC-1 (Sigma-Aldrich, St. Louis, MO, USA), a lipophilic cation that selectively enters the mitochondria and reversibly alters its aggregation state in response to changes in mitochondrial potential. JC-1 shifts from a green monomeric form to orange/red aggregates when the membrane potential increases. Detached cells were stained according to the manufacturer’s recommendations, and the green monomers (FL1) and red aggregates (FL2) were detected by BD FACSAria III flow cytometer. Results were presented as the fold change in the ratio of green to red fluorescence (mean FL1/FL2, arbitrarily set to 1 for control samples), with the increase in FL1/FL2 reflecting mitochondrial hyperpolarization.

### 4.10. Measurement of ROS Accumulation

Total intracellular ROS accumulation was assessed by measuring the fluorescence of cells stained with the redox-sensitive dye dihydrorhodamine 123 (DHR) according to the manufacturer’s recommendations (Thermo Fisher Scientific, Waltham, MA, USA). The mean intensity of green fluorescence (FL1), which corresponds to the total ROS concentration, was determined using a BD FACSAria III flow cytometer. The results were expressed in single-parameter histograms (number of events vs. FL1 intensity).

### 4.11. TBARS Assay

The levels of malondialdehyde (MDA), a by-product of lipid peroxidation, were determined using a colorimetric thiobarbituric acid reactive substances (TBARS) assay. Malondialdehyde binds to thiobarbituric acid (TBA) during incubation and forms a red-pink chromogenic TBARS complex. In brief, the supernatant obtained after centrifugation of cells lysed in 10% ice-cold trichloroacetic acid was mixed with an equal volume of 0.6% TBA and heated in boiling water for 10 min. The intensity of the color developed, which corresponds to the level of lipid peroxidation, was measured spectrophotometrically at 535 nm. The index of lipid peroxidation (ILP) was calculated as the fold change in the absorbance intensity of treated cells compared to the untreated cells, with the control arbitrarily set to 1.

### 4.12. Antiradical Activity Assessment

DPPH Radical Scavenging Assay. Antioxidant activity was measured using the DPPH assay. The reduction in the stable free radical 2,2-diphenyl-1-picrylhydrazyl (DPPH) in the presence of antioxidants results in the loss of the purple DPPH color. Ascorbic acid (AA) was used as a standard antioxidant compound. Briefly, different concentrations of EE, GC, and AA were dissolved in TRIS buffer (pH 7.4) and mixed with a methanol solution of DPPH (50 µM) (1:1). The absorbance of DPPH remaining in the solution after a 30 min incubation was measured at 517 nm using an automated microplate reader. The results were expressed as the percentage of DPPH absorbance in the samples compared to the control, which contained only DPPH and solvent and were considered 100%.

NBT assay. Superoxide scavenging activity was assessed using the nitroblue tetrazolium (NBT) assay, based on the superoxide-dependent conversion of NBT to formazan. Alkaline DMSO (1 mM NaOH/DMSO) was used as a superoxide-generating system, while NAC was used as a standard antioxidant. Different concentrations of EE, GC, or NAC were mixed with alkaline DMSO and NBT solution (0.1 mg/mL). The absorbance of the yielded color, reflecting the amount of superoxide produced, was measured at 560 nm using an automated microplate reader. The results of the NBT assays were expressed as the percentage of superoxide formation compared to superoxide formation in the control (alkaline DMSO alone), which was considered to be 100%.

### 4.13. Conjugated Diene Formation

The influence of EE and GC on the Cu^2+^-induced LDL oxidation process was determined by kinetic measurements of the formation of conjugated dienes, the early products of LDL oxidation. In brief, native LDL (0.1 mg/mL) was exposed to CuSO_4_ (final concentration 10 μM) in the absence or presence of various concentrations of EE or GC. Vitamin E was used as a standard antioxidant compound. The formation of conjugated dienes was monitored using a spectrophotometer (Thermo Evolution 600, Thermo Fisher Scientific, Waltham, MA, USA) at 234 nm every 10 min for 4 h. The lag phase is the period between the addition of Cu²⁺ to LDL and the time at which the conjugated dienes begin to increase. The results are presented as representative plots, and the effect of vitamin E, EE, and GC on LDL oxidation was expressed as a delay in the lag phase compared to LDL treated with CuSO_4_ alone.

### 4.14. Measurement of NO Production

NO production was determined by measuring the accumulation of nitrite, the NO end-product, using the Griess assay. Briefly, the cell culture supernatants were mixed with an equal volume of Griess reagent (1:1 mixture of 0.1% naphthylethylenediamine dihydrochloride and 1% sulfanilamide in 5% H_3_PO_4_) in a flat-bottom 96-well plate and incubated for 10 min. The nitrite concentration was calculated using a NaNO_2_ standard curve. The absorbance of the yielded color, reflecting the level of accumulated nitrite, was measured at 570 nm using an automatic microplate reader. The results were expressed as a fold increase in nitrite accumulation compared to nitrite accumulation in the control, which was arbitrarily set to 1.

### 4.15. Immunoblot

Immunoblotting was used to assess the activity of specific proteins. After washing out dead cells with ice-cold PBS, remaining adherent cells were lysed in RIPA buffer (50 mM Tris, pH 7.5, 150 mM NaCl, 1% Nonidet NP40, 0.1% SDS, 0.5% Triton X100, 1 mM EDTA, 1 mM EGTA, 2 mM DTT with protease and phosphatase inhibitors) for 30 min. The cell lysates were centrifuged at 14,000× *g* for 15 min at 4 °C, and the supernatant was collected. The concentration of isolated proteins was determined by the Lowry method, using bovine serum albumin (BSA) as a standard. Equal amounts of proteins (30–50 µg) from each sample were separated by SDS-PAGE and transferred to PVDF membranes (Immobilon-FL, Merck, Darmstadt, Germany). The membranes were blocked for one hour with 5% non-fat dry milk in PBS at room temperature, and then incubated overnight at 4 °C with the following primary rabbit anti-human antibodies: anti-ERK (#2507s, Cell Signaling Technology, Danvers, MA, USA; 1:1000), anti-phospho-ERK (#45737s Cell Signaling Technology; 1:1000), anti-AMPK (sc-25792, Santa Cruz Biotechnology, Dallas, TX, USA; 1:1000), anti-phospho-AMPK (#2382s, Cell Signaling Technology; 1:1000), anti-CREB (#9197s, Cell Signaling Technology; 1:1000), anti-phospho-CREB (#9198s, Cell Signaling Technology; 1:1000), anti-Akt (#9272s, Cell Signaling Technology; 1:1000), anti-phospho-Akt (#4060s, Cell Signaling Technology; 1:1000), and anti-mouse anti-eNOS (#610296, BD Biosciences, San Jose, CA, USA; 1:1000). Antibodies against β-actin (PA1-183, Thermo Fisher Scientific, Waltham, MA, USA; 1:2000) and GAPDH (#2118, Cell Signaling Technology; 1:10,000) were used as an equal loading controls. After incubation with the primary antibody, membranes were washed extensively and incubated for 90 min with a horseradish peroxidase-conjugated anti-rabbit secondary antibody (#7074, Cell Signaling Technology; 1:2000) or a horseradish peroxidase-conjugated anti-mouse secondary antibody (#7076, Cell Signaling Technology; 1:2000). The specific protein bands were visualized by chemiluminescence using the iBright FL1500 Imaging System (Thermo Fisher Scientific, Waltham, MA, USA). Signal intensity was quantified by densitometry using ImageJ software v1.51j8 (https://imagej.nih.gov/ij/, National Institutes of Health, Bethesda, MA, USA), and the ratio between phosphorylated and corresponding total protein signals was calculated. The results were presented relative to the signal intensity of the untreated control, which was arbitrarily set to 1.

### 4.16. Statistical Analysis

All values are presented as means ± SD. Data were statistically analyzed using *t*-test or one-way ANOVA followed by the Student–Newman–Keuls test. A value of p less than 0.05 was considered statistically significant.

## 5. Conclusions

In conclusion, the results of this study demonstrate that EE from stemless gentian and its main constituent xanthone GC effectively counteract oxLDL-triggered endothelial damage through antioxidant and anti-apoptotic actions. The protection is associated with the restoration of mitochondrial function and the activity of important pro-survival signals—Akt/CREB/eNOS axis and ERK kinase. An ability to delay LDL oxidation and enhance NO bioavailability in oxLDL-exposed cells implies favorable effects of EE and GC that could possibly reduce the risk of adverse cardiovascular events. Therefore, in addition to the previously reported endothelium-independent vasorelaxant potential of *G. kochiana*, our study reveals endothelial-dependent protective effects of this plant and highlights it as a significant source of potentially valuable xanthones for the prevention of cardiovascular diseases.

## Figures and Tables

**Table 1:** The influence of EE, GC, and vitamin E on Cu^2+^-induced LDL oxidation.

	Concentration	Lag Phase Delay (%)
	10 μg/mL	n.d.
EE	5 μg/mL	136.4
	2.5 μg/mL	81.8
	10 μM	163.3
GC	5 μM	133.3
	2.5 μM	83.3
Vit E	10 μM	21.1

n.d.—not determined.

## Data Availability

The original contributions presented in this study are included in the article/Supplementary Material. Further inquiries can be directed to the corresponding author(s).

## Supplementary Materials

The following supporting information can be downloaded at: https://www.mdpi.com/article/10.3390/ijms26031351/s1.
