# Supplementary material for: Endothelial-Protective Actions of Diethylether Extract from Gentiana kochiana and Xanthone Gentiacaulein Against Oxidized LDL-Induced Injury—In Vitro Evaluation
Source: Int J Mol Sci. 2025 Feb 5;26(3):1351. doi: 10.3390/ijms26031351 (PMC11818938; doi:10.3390/ijms26031351)
Supplement: Supplementary file 1 [file ijms-26-01351-s001.zip › ijms-3454579-supplementary.pdf]

Endothelial-Protective Actions of Diethylether Extract from *Gentiana kochiana* and Xanthone Gentiacaulein Against Oxidized LDL-Induced Injury – In Vitro Evaluation

Gordana Tovilović-Kovačević[1,*], Nevena Zogović[2], Đurđica Ignjatović[1], Mirko Tomić[1], Jelena Penjišević[3], Jelena Kukić-Marković[4] and Dijana Krstić-Milošević[5,*]

[1] Department of Biochemistry, Institute for Biological Research "Siniša Stanković"– National Institute of the Republic of Serbia, University of Belgrade, Bulevar despota Stefana 142, 11108 Belgrade, Serbia; tovilovicg@ibiss.bg.ac.rs, djurdjica@ibiss.bg.ac.rs, mitomic@ibiss.bg.ac.rs

[2] Department of Neurophysiology, Institute for Biological Research "Siniša Stanković"– National Institute of the Republic of Serbia, University of Belgrade, Bulevar despota Stefana 142, 11108 Belgrade, Serbia; nevenar@ibiss.bg.ac.rs

[3] Institute for Chemistry, Metallurgy and Technology – National Institute of the Republic of Serbia, University of Belgrade, Njegoševa 12, 11000 Belgrade, Serbia; jelena.penjisevic@ihtm.bg.ac.rs

[4] Department of Pharmacognosy, University of Belgrade–Faculty of Pharmacy, Vojvode Stepe 450, 11221 Belgrade, Serbia; jelena.kukic@pharmacy.bg.ac.rs

[5] Department of Plant Physiology, Institute for Biological Research "Siniša Stanković"– National Institute of the Republic of Serbia, University of Belgrade, Bulevar despota Stefana 142, 11108 Belgrade, Serbia; dijana@ibiss.bg.ac.rs

* Correspondence: tovilovicg@ibiss.bg.ac.rs (G.T.K.); dijana@ibiss.bg.ac.rs (D.K.M.); Tel.: +381-11-2078-305 (G.T.K.); +381-11-2078-394 (D.K.M.)

Supplementary information

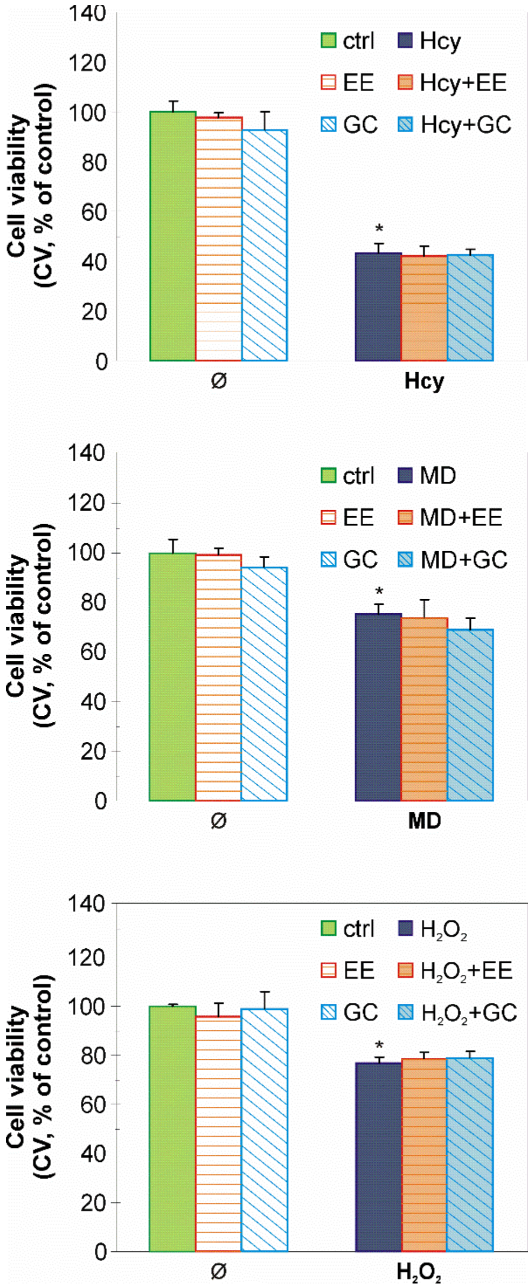

Figure S1. The effect of EE and GC on viability of EA.hy926 cells treated with homocysteine, menadione, and hydrogen peroxide. EA.hy9260 cells were pre-treated with EE (10 µg/ml) or GC (10 µM) for 30 minutes and then exposed to homocysteine (Hcy, 10 mM), menadione (MD, 20 µM), or hydrogen peroxide (H_2O_2, 25 µM) for additional 24 h. Cell viability was assessed using crystal violet test. The results are presented as mean ± SD values of triplicates from one representative out of three independent experiments. * $p < 0.05$ compared to control, untreated cells.
